# Laparoscopic Transhiatal Resection of an Esophageal Diverticulum in a Patient With Systemic Lupus Erythematosus: A Case Report

**DOI:** 10.7759/cureus.68120

**Published:** 2024-08-29

**Authors:** Takaomi Ozawa, Katsutoshi Shoda, Yoshihiko Kawaguchi, Suguru Maruyama, Yudai Higuchi, Ryo Saito, Yuki Nakata, Koichi Takiguchi, Kensuke Shiraishi, Shinji Furuya, Hidetake Amemiya, Hiromichi Kawaida, Daisuke Ichikawa

**Affiliations:** 1 First Department of Surgery, University of Yamanashi, Yamanashi, JPN

**Keywords:** laparoscopic approach, pseudodiverticulum, minimally invasive surgery, systemic lupus erythematosus, epiphrenic diverticula, esophageal diverticula

## Abstract

Esophageal diverticula are relatively uncommon, especially supradiaphragmatic diverticula. Esophageal diverticula are normally managed by observation; however, surgical treatment is sometimes indicated for large diverticula or diverticula in highly symptomatic patients. Surgical approaches for esophageal diverticula include thoracoscopic or laparoscopic resection; however, consensus has not yet been reached on the optimal approach. Here, we report a case of safe laparoscopic transhiatal esophageal diverticulectomy in a patient with a giant esophageal diverticulum with severe coexisting disease.

The patient was a 63-year-old woman with a 17-year history of systemic lupus erythematosus (SLE) who was managed by outpatient therapy with steroids and immunosuppressive drugs. She had a history of SLE-associated renal dysfunction and SLE-associated pulmonary artery thromboembolism, and she was receiving anticoagulation therapy. During an outpatient visit, the patient experienced pericardial discomfort, and upper gastrointestinal endoscopy and computed tomography revealed the presence of a diaphragmatic diverticulum with a diameter of 3 cm. She subsequently developed aspiration pneumonia, which was thought to be caused in part by food stagnation in the diverticulum. However, due to the risks associated with systemic complications, she was initially managed by observation. One year later, the diverticulum had expanded to 6 cm in diameter, and it was determined that the risk of esophageal perforation and aspiration pneumonia was high. Surgery was performed under a laparoscope, and the diverticulum was resected with surgical staplers under an extremely good visual field by dissecting the area around the esophageal hiatus. Postoperative pathology confirmed that the diverticulum was a pseudodiverticulum. The patient’s postoperative course was initially good, and she was discharged 10 days after surgery. However, the day after discharge, a hematoma infection occurred near the suture site, requiring re-hospitalization and drainage surgery. After reoperation, she recovered without complications and was discharged 14 days later. Subsequent follow-up showed no diverticulum or pneumonia recurrence.

The laparoscopic approach is a minimally invasive approach for patients with diverticula who are at high surgical risk. With an adequate view from the abdominal cavity, even a patient with a fairly large diverticulum can be safely resected.

## Introduction

Esophageal diverticula are classified into pharyngoesophageal, mid-esophageal, and epiphrenic diverticula based on their location of occurrence. Zenker’s diverticulum, which arises in the pharyngoesophageal region, is the most common type, followed by epiphrenic diverticula located above the diaphragm [1,2]. The frequency of epiphrenic diverticula detection in upper gastrointestinal contrast studies is reported to be 0.9%, indicating its rarity [3]. Many cases of epiphrenic diverticula are associated with esophageal achalasia and often form a pulsion pseudodiverticulum on the right side of the esophagus [2]. Although observation is often considered for management, surgery is indicated according to the diagnostic criteria proposed by Goodman in patients who present with either symptomatic diverticula or complications, such as ulcers, malignant tumor complications, or stasis of the diverticular contents [4]. However, there is no consensus on the optimal surgical approach, with options including open thoracotomy, thoracoscopic resection, and esophagectomy using laparoscopy [1,2,5]. Although there is no consensus on the preferred surgical approach for supradiaphragmatic esophageal diverticula, laparoscopic surgery, which is minimally invasive, has become increasingly favored in recent years. However, because of the risk of organ damage due to surrounding adhesions in cases of giant diverticula, thoracoscopic surgery is often chosen to ensure adequate visualization [2].

In this study, we report a case of a patient with a large esophageal epiphrenic diverticulum who experienced repeated pneumonia and had severe coexisting systemic lupus erythematosus (SLE) and underwent safe surgery by laparoscopic surgery.

## Case presentation

**Figure 1:**
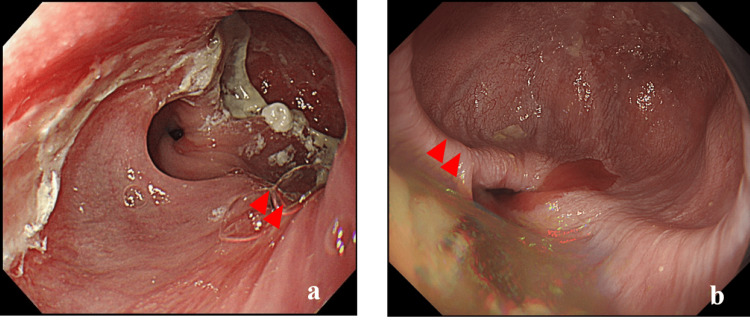
Upper gastrointestinal endoscopy findings (a) A 3-cm (diameter) diverticulum (red arrow) can be seen just above the esophagogastric junction; (b) Upon follow-up one year later, the esophageal diverticulum had expanded from 3 cm to 6 cm.

The patient was a 63-year-old woman with a 17-year history of SLE who was receiving outpatient treatment with steroids and immunosuppressive agents. The patient had a history of SLE-associated renal impairment and pulmonary artery thromboembolism and was being treated with anticoagulation therapy. The patient also had comorbid hypertension and dyslipidemia. During an outpatient visit, the patient experienced pericardial discomfort, and upper gastrointestinal endoscopy and computed tomography (CT) revealed the presence of a diverticulum with a diameter of 3 cm on the diaphragm (Figure 1a). The patient was subsequently referred to our department, where blood tests showed mild renal dysfunction (estimated glomerular filtration rate: 17 mL/min/1.73 m²) and prolonged prothrombin time (prothrombin time/international normalized ratio: 1.5) due to anticoagulation therapy, with no other abnormalities.

Due to the high perioperative risk associated with SLE complications and the patient’s history of steroid and immunosuppressive drug use, the initial plan was for follow-up rather than surgical treatment. However, a follow-up endoscopy one year later revealed a marked increase in the size of the diverticulum to 6 cm in diameter (Figure 1b).

**Figure 2:**
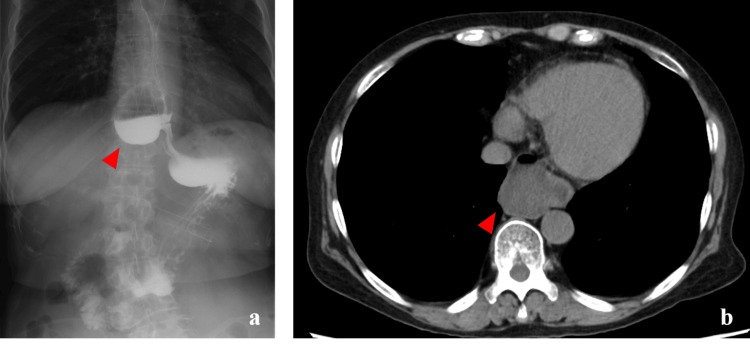
Preoperative imaging findings (a) Preoperative upper gastrointestinal imaging showing a diverticulum on the right side of the thoracic esophagus just above the diaphragm. Even after the contrast agent flowed into the stomach, residual contrast remained within the diverticulum (red arrow); (b) Preoperative CT image showing food residue remaining within the diverticulum on the right side of the esophagus (red arrow). CT: computed tomography

CT and upper gastrointestinal contrast studies indicated contrast retention within the diverticulum (Figure 2).

The patient decided to undergo surgical resection because she had experienced repeated aspiration pneumonia due to the accumulation of food residue in the diverticulum over the past year. Moreover, the diverticulum was growing, so the possibility of future esophageal diverticulum perforation was considered. Among the surgical treatment options, laparoscopic transhiatal esophageal diverticulectomy was planned, which was considered to be the most noninvasive.

**Figure 3:**
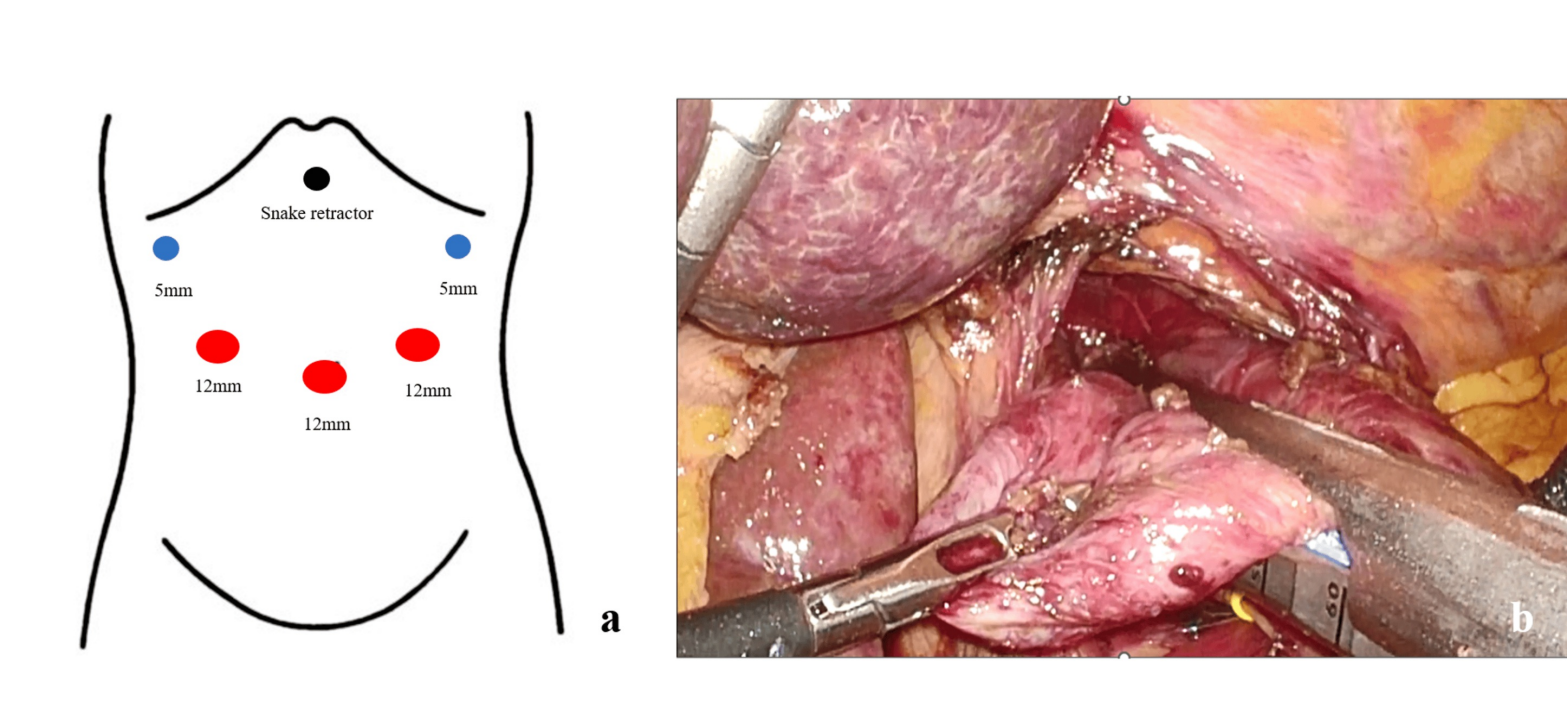
Intraoperative image of laparoscopic transhiatal esophageal diverticulectomy (a) Laparoscopic procedure was performed using five ports, with a snake retractor for liver retraction inserted at the epigastrium midline. A surgical stapler was inserted through the right lower abdominal port; (b) After the periesophageal area was dissected and the entire diverticulum, including the cephalic side, was exposed, the diverticulum was resected with a stapler. The vagus nerve was pre-taped with yellow tape to avoid excision. The angle of the stapler was considered suitable for laparoscopy.

A five-port laparoscopic procedure was performed (Figure 3a). After taping the esophagus, the esophageal hiatus was dissected to ensure adequate space. The diverticulum was completely dissected circumferentially to ensure vagus nerve preservation, which was carefully secured with vessel tape. A surgical stapler was inserted through the right lower abdominal port, and two surgical staplers were used to resect the diverticulum (Figure 3b).

We performed the diverticulectomy with intraoperative endoscopy to check the diverticulum and the esophageal lumen.

After resection, we inspected the mucosal side for any defects or esophageal stenosis. A drain was inserted into the esophageal hiatus site. The esophageal hiatus dissection site was sutured with 3-0 proline sutures, followed by the stomach and diaphragm, also with 3-0 proline sutures. The surgery lasted for 210 minutes, with a blood loss of 19 mL.

**Figure 4:**
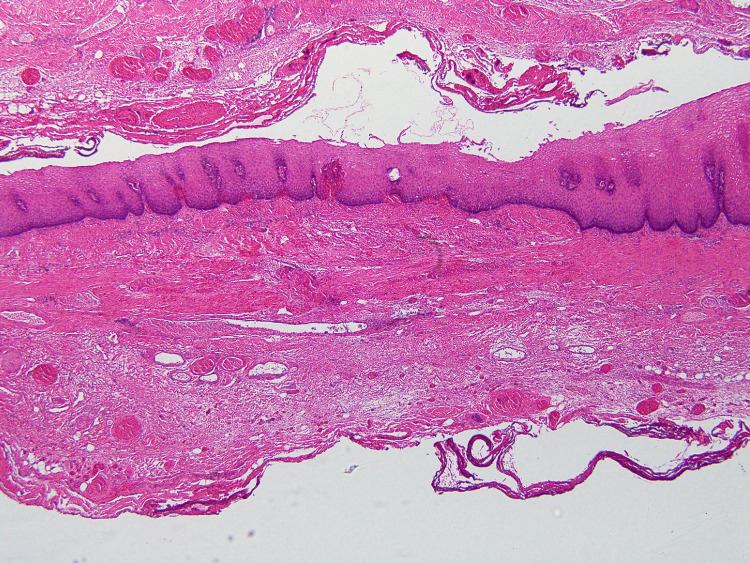
Histological image of the excised esophageal diverticulum (×40) The muscularis propria of the esophagus was nearly absent, leading to the diagnosis of pseudodiverticulum.

Postoperative pathology revealed that the resected specimen was a pseudodiverticulum (Figure 4).

**Figure 5:**
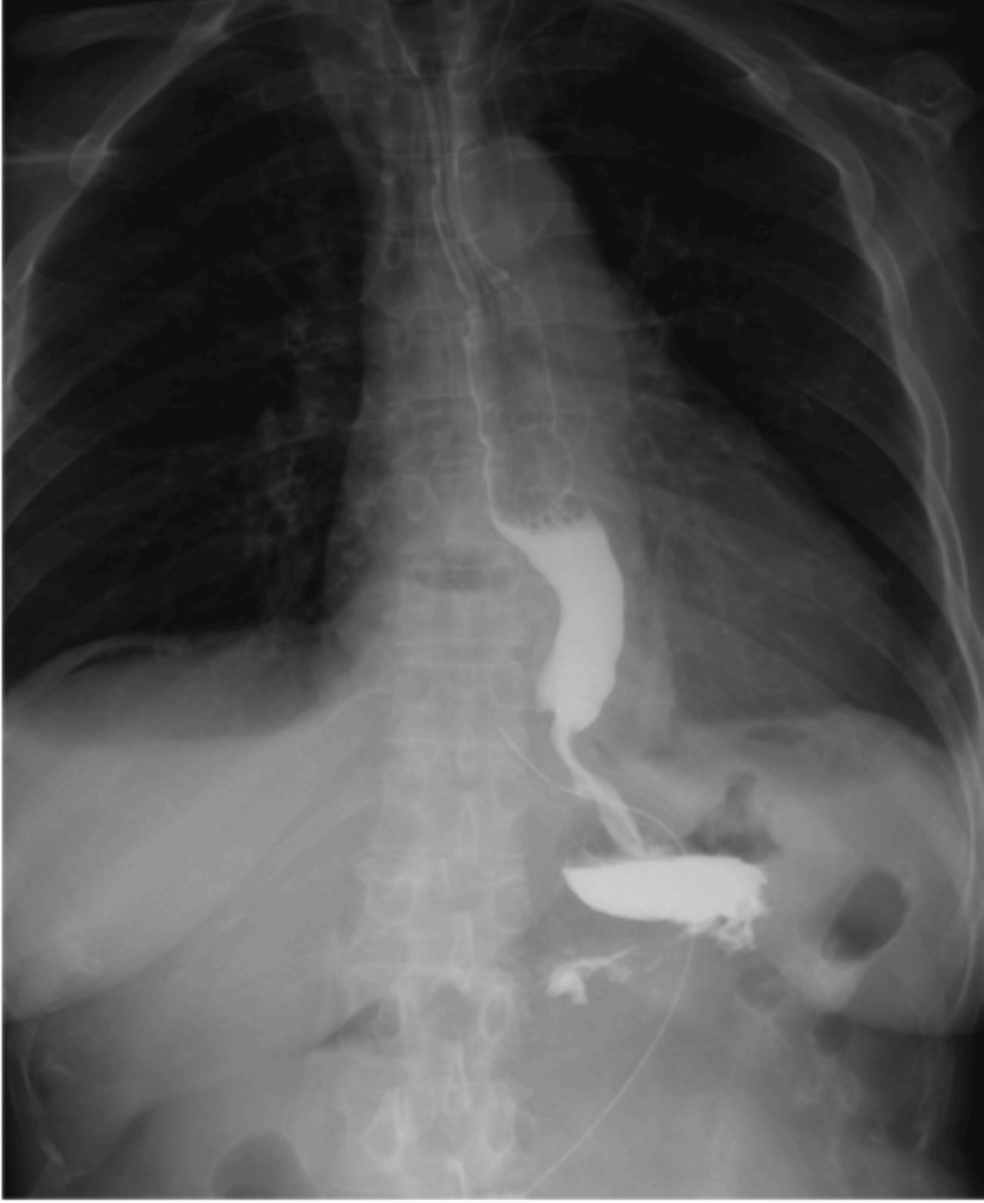
Upper gastrointestinal contrast image one week postoperatively showing the smooth passage of contrast No apparent contrast leakage or esophageal motility disorders were observed.

The patient’s postoperative recovery was smooth, and postoperative upper gastrointestinal contrast studies showed no leakage of contrast medium (Figure 5).

The patient was discharged on postoperative day 10. However, the day after her discharge, the patient developed a hematoma infection near the suture site, requiring readmission for drainage surgery. After reoperation, the patient recovered well and was discharged on postoperative day 14. Subsequent follow-up showed no diverticulum or pneumonia recurrence.

## Discussion

Esophageal diverticula are rare, with an estimated prevalence of 0.06%-3.6% in barium swallow studies [6]. Among the types of esophageal diverticula, supradiaphragmatic diverticula, which are analogous to Zenker’s diverticula, account for approximately 10% of cases [1]. Considering the rarity of esophageal diverticula, their association with SLE is exceptionally uncommon, with only three cases reported previously in the literature [7-9]. Notably, all previously reported cases involved mid-esophageal diverticula, making our case the first report of a diaphragmatic diverticulum associated with SLE.

It is noted that mid-esophageal diverticula and epiphrenic diverticula are associated with esophageal motility disorders, with reports suggesting that 73%-81% of epiphrenic diverticula are accompanied by motility disturbances [2,10,11]. A previous study suggested that approximately 25% of patients with SLE experience esophageal dysmotility [12]. In this case, although preoperative endoscopy and upper gastrointestinal contrast image did not indicate any gross motility disorders, we were unable to perform manometry due to social circumstances, so the possibility of an underlying motility disorder cannot be ruled out. Should the patient develop any symptoms in the future, or if consent for testing is obtained, postoperative esophageal motility testing may be considered. Furthermore, long-term steroid therapy can weaken tissue integrity, potentially contributing to diverticulum formation. Therefore, even in the absence of conditions such as achalasia or peritracheal lymphadenitis, patients with SLE undergoing treatment may still be at risk of diverticulum development.

Regarding treatment, follow-up is often considered, but surgical treatment is the treatment of choice when Goodman’s diagnostic criteria are met [4]. In the present case, the surgical treatment plan was determined based on the patient’s immunocompromised status, which increased the risk of recurrent aspiration pneumonia, worsening respiratory disease, and increased the possibility of esophageal diverticulum perforation [13]. Nevertheless, supradiaphragmatic diverticulectomy comes with its own complications, with leakage from the suture line being the most commonly reported, with an incidence of around 15% [14]. Furthermore, in patients in whom steroid therapy is used, the additional bleeding tendency due to heparin, as well as renal dysfunction due to SLE, may increase the likelihood of complications related to suture line leakage. Therefore, it is imperative to carefully consider the risks posed by both diverticular pneumonia and surgical intervention.

Regarding surgical approaches, laparoscopic or mediastinoscopic resection is often preferred for the treatment of diaphragmatic diverticula because of its minimally invasive nature and the fact that it is less time-consuming due to its simplicity. However, in cases of giant diverticula, a thoracoscopic resection approach is often chosen because of the difficulty in achieving adequate visualization [1,2,15]. Therefore, in cases where adequate intraoperative visualization is difficult, preparation for the thoracoscopic approach or sub-total esophageal dissection with laparoscopy is considered necessary. In this case, diverticulectomy was performed safely, easily, and minimally invasively with good visualization by dissecting the esophageal hiatus. In the present case, a myotomy of the lower esophageal muscular layer, which is sometimes performed in addition to diverticulectomy, was not performed because of the risk of excessive tissue damage associated with steroid therapy and bleeding under anticoagulant therapy [1].

Sufficient exposure obtained by extensive esophageal hiatus dissection greatly facilitated visualization, and, as reported elsewhere, the use of an automated suture device facilitated smooth dissection, especially with the laparoscopic approach [2]. Vertical dissection of the cephalic side of the diaphragmatic crus to create space and adequate traction of the abdominal esophagus near the diverticulum with cotton tape were effective for achieving optimal visualization. Furthermore, the use of an intraoperative endoscope was advantageous in ensuring closure of the muscular layer with surgical staplers, given the nature of the pseudodiverticulum. These modifications suggest that the laparoscopic approach is feasible for large paraesophageal supradiaphragmatic diverticula and may be considered as a first choice, especially in patients with a high surgical risk.

## Conclusions

The esophageal diverticulum associated with SLE is a rare entity; however, it is noteworthy due to its pathophysiologic potential. Laparoscopy for supradiaphragmatic esophageal diverticula is a less invasive approach and can safely dissect even considerably sized diverticula while ensuring adequate visualization.
